# Therapeutic Applications of Terpenes on Inflammatory Diseases

**DOI:** 10.3389/fphar.2021.704197

**Published:** 2021-08-13

**Authors:** María Luisa Del Prado-Audelo, Hernán Cortés, Isaac H. Caballero-Florán, Maykel González-Torres, Lidia Escutia-Guadarrama, Sergio A. Bernal-Chávez, David M. Giraldo-Gomez, Jonathan J. Magaña, Gerardo Leyva-Gómez

**Affiliations:** ^1^ Escuela de Ingeniería y Ciencias, Departamento de Bioingeniería, Instituto Tecnologico de Monterrey, Ciudad de México, México; ^2^ Laboratorio de Medicina Genómica, Departamento de Genómica, Instituto Nacional de Rehabilitación Luis Guillermo Ibarra Ibarra, Ciudad de México, México; ^3^ Departamento de Farmacia, Facultad de Química, Universidad Nacional Autónoma de México, Ciudad de México, México; ^4^ Departamento de Farmacología, Centro de Investigación y de Estudios Avanzados del Instituto Politécnico Nacional, Ciudad de México, México; ^5^ CONACyT-Laboratorio de Biotecnología, Instituto Nacional de Rehabilitación Luis Guillermo Ibarra Ibarra, Ciudad de México, México; ^6^ Departamento de Física, Facultad de Ciencias, Universidad Nacional Autónoma de México, Ciudad de México, México; ^7^ Departamento de Biología Celular y Tisular, Facultad de Medicina, Universidad Nacional Autónoma de México (UNAM), Ciudad de México, México; ^8^ Unidad de Microscopía, Facultad de Medicina, Universidad Nacional Autónoma de México (UNAM), Ciudad de México, México

**Keywords:** terpenes, anti-inflammatory, terpenoids, formulations, natural products

## Abstract

In the last decades, the search for natural products with biological applications as alternative treatments for several inflammatory diseases has increased. In this respect, terpenes are a family of organic compounds obtained mainly from plants and trees, such as tea, cannabis, thyme, and citrus fruits like lemon or mandarin. These molecules present attractive biological properties such as analgesic and anticonvulsant activities. Furthermore, several studies have demonstrated that certain terpenes could reduce inflammation symptoms by decreasing the release of pro-inflammatory cytokines for example, the nuclear transcription factor-kappa B, interleukin 1, and the tumor necrosis factor-alpha. Thus, due to various anti-inflammatory drugs provoking side effects, the search and analysis of novel therapeutics treatments are attractive. In this review, the analysis of terpenes’ chemical structure and their mechanisms in anti-inflammatory functions are addressed. Additionally, we present a general analysis of recent investigations about their applications as an alternative treatment for inflammatory diseases. Furthermore, we focus on terpenes-based nanoformulations and employed dosages to offer a global perspective of the state-of-the-art.

## Introduction

Inflammation is a complex biological response derived from damaging stimuli such as wounds, infections, pathogens, and other foreign substances. In a typical case, the organism attempts to remove harmful stimuli through a meticulous process, initiating the self-healing protective defense against infection (Chong et al., 2019). On the other hand, in some diseases, the immune system produces an inflammatory response in the absence of foreign substances or infection. These heterogeneous diseases are characterized by an overproduction of inflammatory cytokines that include interleukin-1β (IL-1β) and tumor necrosis factor-alpha (TNF-α) (Gu et al., 2012). Synthetic anti-inflammatory compounds have been widely employed for suppressing or inhibiting these mediators. However, side effects are commonly related to their application. For this reason, in recent years, natural products have been explored as an alternative drug delivery treatment with safe toxicological profiles.

In this context, terpenes are a highly diverse family of natural products which are synthesized by plants. This family have approximately 55,000 members with different chemical structures, presenting potential practical applications (Prakash, 2017; Serrano Vega et al., 2018). For this reason, it has been reported that terpenoids could ameliorate various symptoms caused by inflammation, inhibiting various steps of inflammatory processes. However, due to their low solubility and high instability, some alternatives, such as nanotechnology, have been explored.

This article aims to provide a comprehensive short review of this class of compounds and their potential application as an anti-inflammatory treatment therapy. Furthermore, we mention the nanoformulations and dosages of these compounds to evaluate differences triggered by these conditions and offer a global perspective.

## Physicochemical Information

**FIGURE 1:**
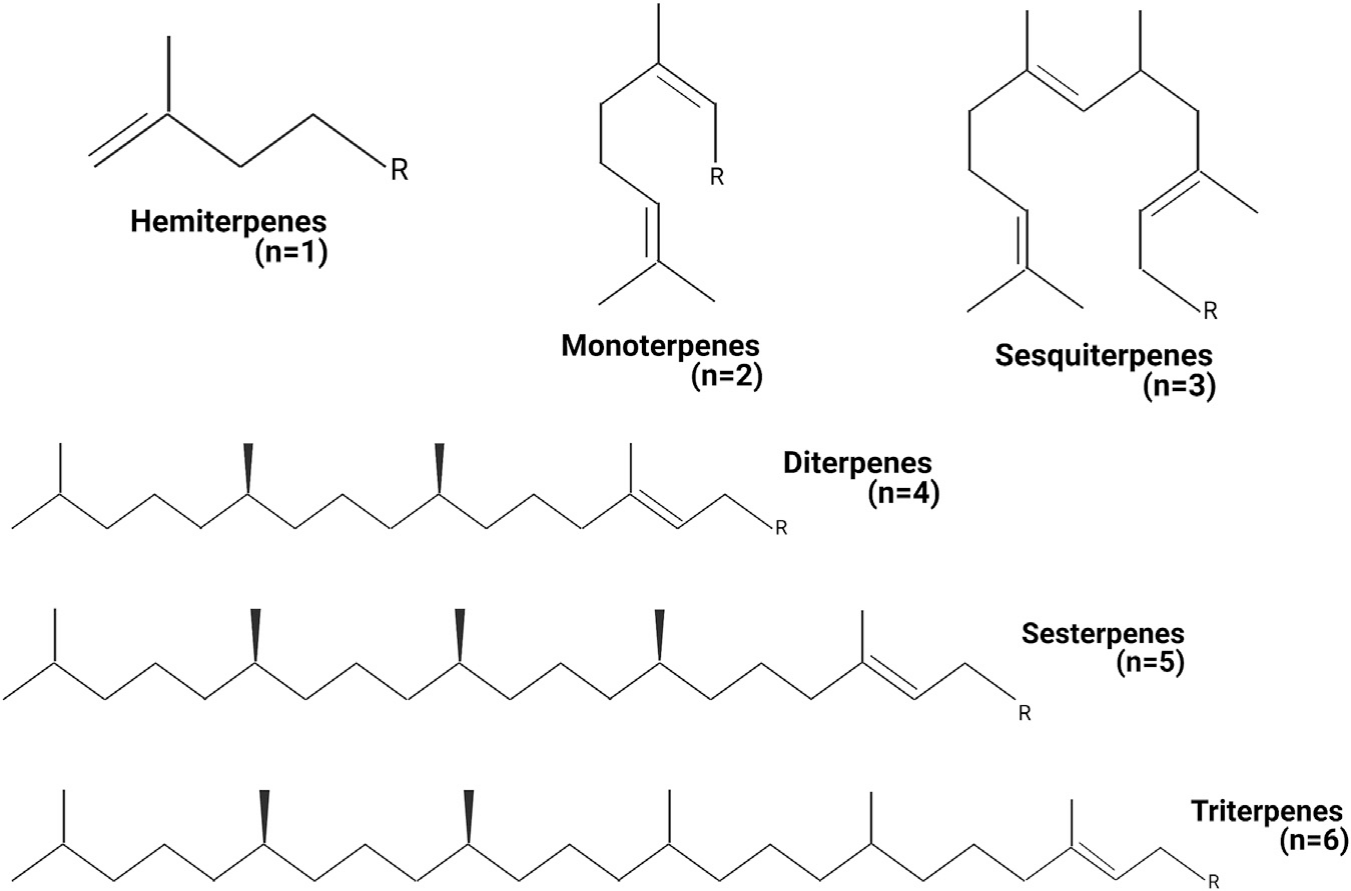
Classification of terpenes. The terpenes can be classified by the number of units of isoprene present in the chemical structure. n = amount of isoprene units in the different terpene structures. Created with BioRender.com.

Terpenes have the general chemical formula (C_5_H_8_)_n_, defined by the isoprene as a unit. Nevertheless, not all terpenes have even numbers of intact isoprene units, and some of them are different, such as the C_19_ diterpenoids. Terpenes are described as terpenoids when functional groups as alcohols, aldehydes, or ketones are present in their chemical structure. One of the most applied classifications for terpenoids is based on the number of isoprene units (Figure 1). Monoterpenoids have a chemical structure based on two isoprene units (C_10_H_16_) and express different arrangements as acyclic, monocyclic, and bicyclic. Sesquiterpenoids present three isoprene units (C_15_H_24_), often structured simple to complex mono- and polycyclic rings. Triterpenoids (C_30_H_48_) have a wide distribution from more than 40 different carbon skeletons. Tetraterpenoids (C_40_H_64_) have a large structure, and they are also known as carotenes. Nowadays, around 1,000 monoterpenes, 7,000 sesquiterpenes, and 3,000 diterpenes have been described, and the list increases year by year. The biosynthetic pathway for terpenes is based on the well-known malonic acid pathway and, possibly, by the non-mevalonate pathway consuming triose phosphate (Zwenger and Basu, 2008; Brahmkshatriya and Brahmkshatriya, 2013).

Each terpene needs a purification protocol by the different chemical properties. In this respect, Jiang et al. reported three basic modes to extract terpenes, i) based on the non-polar terpenes, ii) terpenoids with low polarity, and iii) polar terpenoids (Jiang et al., 2016).

Terpenes are biosynthesized in superior plants or *Spermatophytes* (plants that produce seeds) from isopentyl pyrophosphate and its isomer dimethylallyl pyrophosphate through the plastidic methylerythritol phosphate and the cytosolic mevalonate pathways. Along the biosynthetic pathways, the terpene synthase is the critical enzyme that catalyzes the formation of hemi-, mono-, sesqui-, or diterpenes. Moreover, the vast types of terpenoids arise from the different terpene synthase types present in the plants (Liu et al., 2020). However, recently some groups have analyzed synthetic alternative reactions to obtain complex terpenoids *in vitro*. For example, Trost and Min explored a synthesis for terpenes cyclization catalyzed with palladium. The polyenyne cycloisomerization revealed an efficient and scalable synthesis of tremulanes. Moreover, the authors reported an excellent functional group tolerance, stereocontrol, and a perfect atom economy (Trost and Min, 2020).

## Anti-inflammatory Activity of Terpenes Pathways

The inflammation process represents a cascade of dynamic responses, including cellular and vascular actions with specific humoral secretions (Huether and McCance, 2016; Abdulkhaleq et al., 2018). Acute and chronic inflammatory diseases cause excessive or long-lasting tissue damages. These conditions are promoted by the overproduction of pro-inflammatory cytokines and other inflammatory mediators, such as IL-1, IL-6, TNF-α, nitric oxide (NO) synthesized by inducible NO synthase, and prostaglandin E2 synthesized by cyclooxygenase-2 (COX-2) (Kim et al., 2020). Likewise, the nuclear factor-κB (NF-κB) is a central transcription factor that regulates pro-inflammatory genes’ expression during inflammation (Lawrence, 2009; Kim et al., 2020). Indeed, deregulated NF-κB expression is a characteristic phenomenon in several inflammatory diseases; thus, NF-κB has become a significant target in drug discovery (George, 2006; Prakash, 2017).

Numerous isolated bioactive terpenes compounds have shown the potential to reduce inflammation by broad mechanisms. Next, we discuss some routes through which terpenes generate these outcomes.

### Effect of Terpenes in Pro-inflammatory Molecules

In the inflammation pathway, triggered by pro-inflammatory cytokines such as IL-1 and TNF-α, the activated NF-κB translocates to the nucleus, and stimulates the production of pro-inflammatory genes (Lawrence, 2009). The general effect of the terpenes in most studies is reducing the pro-inflammatory cytokines expression. For example, some terpenes (D-Limonene, α-Phellandrene, Terpinolene, Borneol, Linalool, and triterpene glycosides) can reduce the expression of TNF-α, IL-1, and IL-6 in the Raw 264.7 macrophages cell line (Yoon et al., 2010; Huo et al., 2013; de Christo Scherer et al., 2019; Li et al., 2019). Similar results were obtained in experiments using *in vivo* models such as swiss mice, Wistar rats, and albino mice (BALB/C) (Ramalho et al., 2015; Li et al., 2016; Gonçalves et al., 2020). The authors consider that the inhibitory effects involve the inhibition of the NF-κB signaling pathway. However, they could not exclude the involvement of other transcription factors. Therefore, despite the studies done in identifying several terpenes in different *in vitro* and *in vivo* models, the mechanism of action underlying the reduction of pro-inflammatory cytokines expression effects is not entirely understood and should be evaluated in further studies.

### Effect of Terpenes on Inflammatory Mediators

The pro-inflammatory mediators activate the signal transduction pathways of the core mitogen-activated protein kinases (MAPKs) such as extracellular signal-regulated kinase, c-Jun N-terminal kinase, and p38, which, in turn, control various cellular processes (Kyriakis and Avruch, 2012). Diverse studies suggested that the terpenes α-pinene, D-limonene, and myrcene decrease the expression of these kinases in mouse peritoneal macrophages, in albino mice, and in human chondrocytes (Chi et al., 2013; Kim et al., 2015; Rufino et al., 2015). Concerning the pathway to reducing the inflammation cascade, the studies agreed on the critical role of the NF-κB. The heterodimers of NF-κB components, mostly p50/p65, are usually retained in the cytoplasm in an inactive form by being associated with an inhibitor of κB protein (IκB) (Baeuerle and Baltimore, 1996; Chi et al., 2013). A wide variety of stimuli can cause the phosphorylation of IκB, a process that is followed by the protein’s ubiquitination and subsequent degradation. The phosphorylation-induced degradation of the IκB enables the NF-κB dimers to enter the nucleus and activate specific target gene expression (Gilmore, 2006; Chi et al., 2013). In this regard, Chi et al. reported that the limonene pre-treatment inhibited pro-inflammatory cytokines and, remarkably, blocked the phosphorylation of IκB in the lipopolysaccharide-induced damage model. The authors suggested that the limonene protection could be mediated by the inhibition of NF-κB (Chi et al., 2013).

### Autophagy

Autophagy has an essential role in pathological conditions such as Parkinson’s disease, Alzheimer’s disease, Crohn’s disease, and cancer (Ashrafizadeh et al., 2020). Autophagy is the self-digestion process where cells use lysosomes to degrade their macromolecules and organelles (Cao et al., 2017; Fornai and Puglisi-Allegra, 2021). Monoterpenes can both downregulate and upregulate autophagy by affecting the mammalian target of rapamycin (mTOR), adenine monophosphate-activated protein kinase, and autophagy-related genes and proteins (ATG). The molecular mechanism of autophagy involves ATGs such as Beclin-1, responsible for the nucleation and expansion of the autophagosome and the microtubule-associated protein light chain 3 (LC3), recruited to the autophagosome double-membrane through an ATG5-dependent mechanism (Fernandes, 2015). For example, geraniol induces neuroprotective activity by inhibiting mTOR and activation of mitophagy and ATG6. Likewise, citral has demonstrated great autophagy stimulation through LC3B and ATG5 upregulation, which results in a potential inhibition of tumor growth (Ashrafizadeh et al., 2020). Some other terpenes with potential activity on autophagy are terpinene-4-ol and α-phellandrene, which act *via* the activation of LC3-I/II. Furthermore, borneol, β-elemene (Nuutinen, 2018), carvacrol (Ashrafizadeh et al., 2020), cucurbitacin E (Stacchiotti and Corsetti, 2020), p-cymene, and camphor (Kim et al., 2020) can increase the autophagy activity. On the other hand, Russo et al. indicated that bergamot essential oil increased the lipidated protein light chain 3 and enhanced autophagy triggered by serum starvation rapamycin. The activity was associated with the two most abundant monoterpenes found in the essential oil, d-limonene and linalyl acetate (Russo et al., 2014).

### Effect on Reactive Oxidative Species

Reactive oxygen species (ROS) can also indirectly modulate the autophagy machinery (Fornai and Puglisi-Allegra, 2021). Due to their antioxidant behavior, terpenes have been shown to protect against different diseases, including neurodegenerative and cardiovascular diseases, cancer, diabetes, and aging processes (Gonzalez-Burgos and Gomez-Serranillos, 2012). Some terpenes with activity on the oxidative stress are α-pinene, d-limonene, camphene, myrcene, p-cymene, terpinolene, camphor, linalool, humulene, β-caryophyllene. Selected mechanisms of action of terpenes on ROS involves the decrease of lipid peroxidation induced by H_2_O_2_, ROS formation, NO release, transforming growth factor (TFG)-1 and type I procollagen secretions, phosphorylation of various MAPK-related signaling molecules, O_2_-production, and H_2_O_2_-induced astrocytic cell death. Furthermore, terpenes can increase catalase, superoxide dismutase, peroxidase activities, and reduced glutathione content, and restore the mitochondrial membrane (Kim et al., 2020).

### Other Pathways

Terpenes such as α-pinene, 3-carene, limonene, and β-caryophyllene directly bind to gamma-aminobutyric acid receptors, decreasing the acetylcholinesterase and lipoxygenase activities, G2/M-phase cell cycle arrest, among others (Kim et al., 2020). For example, Li et al. (2015) suggested that linalool inhibits lipopolysaccharide-induced inflammation in microglial cells, not only by the NF-κB pathway but also by activating factor erythroid 2-related factor/heme oxygenase-1 signaling pathway. On the other side, linalool was also able to upregulate the expression of cell cycle inhibitors in leukemia cells after 12 h. Likewise, citral can induce cell arrest in the G2 phase in breast cancer cells (Fernandes, 2015).

## Therapeutic Potentials on Inflammatory Diseases

Terpenes have been employed as an alternative treatment in inflammatory diseases such as asthma (Lemanske and Busse, 2010), arthritis (Xin et al., 2019; Kim et al., 2020; Singh et al., 2020; Zhang and Wei, 2020), skin inflammation and neuroinflammation. In this section, we focus in therapeutic application of these compounds in skin- and neuroinflammation, in order to present deeper information about the treatments.

### Neuroinflammation

Inflammation is a typical pathological feature involved in the progression of neurodegenerative diseases, such as Alzheimer’s disease and Parkinson’s disease (Amor et al., 2014). Microglia are the resident macrophage cells in the central nervous system and host defense mechanisms producing pro-inflammatory cytokines and ROS (Lee et al., 2018). When activated during brain injuries or by invading pathogens, they produce pro-inflammatory cytokines such as IL-1β, IL-16, TNFα, and TGF-β1 (Fu et al., 2018). Studies reveal that terpenes and terpenoids are involved in suppressing microglia-mediated inflammation involved in acute or chronic neurodegenerative diseases (Sundaram and Gowtham, 2012; Ong et al., 2015; Quintans et al., 2019). In this context, different authors have evaluated the anti-inflammatory and antioxidant effects of the monoterpene Linalool by *in vitro* and *in vivo* studies (Batista et al., 2010; Li et al., 2015). Results strongly suggested this molecule’s neuroprotective activity, acting in NF-κB activation and preventing its nuclear translocation.

D-limonene is one of the most common terpenes in nature and is also involved in neuroinflammation regulation process (Erasto and Viljoen, 2008). D’Alessio et al. have demonstrated that limonene reduced the inflammatory response and decreased the levels of inflammatory cytokines such as IL-1, IL-6 and TNF-α, which are associated with depression (Leonard, 2007; D’Alessio et al., 2014). Also, Lorigooini et al. observed that limonene exerted anti-depressant like effects in maternal separation mice due to the reduction of nitrite levels in the hippocampus (Lorigooini et al., 2021).

In addition, other authors have reported Ginkgolides’ therapeutical effects, the main group of terpenoids from the *Ginko Biloba* tree. Their antioxidant, anti-inflammatory, and neuroprotective activities are due to the downregulation of the Toll-like-receptor 4/NF-κB pathway (Gu et al., 2012).

### Skin Inflammation

Inflammatory skin diseases have a high prevalence, and their treatment is of great interest in the medical community. Hence, multiple studies have been conducted to determine the therapeutic role of terpenes in skin disorders. Notably, the potential of topically administered terpenes due to their anti-inflammatory action has been investigated (Lasoń, 2020). For instance, tea tree oil regulates the edema associated with the efferent phase of a contact hypersensitivity response in mice (Brand et al., 2002a). Additionally, tea tree oil and terpinen-4-ol reduce the skin edema caused by the histamine injection (Brand et al., 2002b; Carson et al., 2006). Likewise, some studies have suggested that thymol (a component of thyme oil) possesses the potential for treating inflammatory processes in the skin (Pivetta et al., 2018); it also exhibits antibacterial, antioxidant, and anesthetic activity (Haeseler et al., 2002; Braga et al., 2006; Wattanasatcha et al., 2012). Furthermore, thymol is useful in acute skin inflammation because it has proven effectiveness with pro-inflammatory models, such as arachidonic acid, histamine, and crotol oil (Veras et al., 2013). In addition to this, Mei et al. (2005) reported that triptolide could significantly suppress the carrageenan-induced edema in the footpad of the rat.

Moreover, α-pinene, which is found in essential oils of coniferous trees, has been studied due to its photoprotective effect against inflammatory signaling in human skin epidermal keratinocytes (Karthikeyan et al., 2018). The authors determined that α-pinene prevents UVA-induced inflammatory protein expression such as TNF-α and IL-6. In addition, it was suggested that the anti-inflammatory activity of α-pinene is due to the suppression of MAPKs pathway through inhibition of ERK and JNK phosphorylation (Kim et al., 2015).

## Terpenes Side Effects

Despite their anti-inflammatory activity, terpenes and terpenoid compounds exhibit low solubility and bioavailability, reduced cell penetration, and also high instability related to their high volatility, representing major difficulties in achieving sustained effects (El-hammadi et al., 2021). For this reason, topical terpenoids alone are not the standard presentation of administration, even when they have been used as enhancers in transdermal formulations for facilitating penetration of other drugs (Aqil et al., 2007).

## Terpenes and Nanoformulations

Nanotechnology represents a strategy to ameliorate the plant extracts complications. These nanocarriers promote sustained release of bioactive molecules, reduce the required dosage, improve the biodistribution, enhance the solubility, all of these potentiating the plant's extracts action (Bonifácio et al., 2014; Fonseca-Santos et al., 2015). In this context, the encapsulation of terpenes in nanostructures is an attractive alternative. For example, in 2018, Pivetta et al. employed nanostructured lipid carriers (NLC) to encapsulate thymol, which presents antimicrobial, antioxidant, and antiseptic properties (Pivetta et al., 2018). The NLC anti-inflammatory activity was evaluated using the cutaneous acute inflammation model. For this purpose, gel formulations were employed for the NLC incorporation achieving a topical administration. The results suggested that the thymol-NLC-gel presented higher edema inhibition compared to the free thymol. Similarly, a study based on lupeol demonstrated that the lowest doses of a lupeol-loaded nanosystem (5 µg) presented higher inflammatory effects than the non-encapsulated lupeol at the highest dose (Cháirez-Ramírez et al., 2015). On the other hand, Bano et al. explored the lycopene impacts on skin edema and inflammation (Bano et al., 2020). Topical pre-treatments of lycopene (10 µg) and lycopene-loaded nanoparticles at two doses (0.5–1 µg) presented a cytoprotective effect; moreover, nanoparticles significantly inhibited the COX-2 expression.

Terpenes such as thymoquinone has been encapsulated and tested in neurological pathologies (Ahmad et al., 2016; Ramachandran and Thangarajan, 2016). Ramachandran S. et al. demonstrated that solid lipid nanoparticles attenuated the levels of oxidative stress and inflammation markers. This behavior is related to thymoquinone ability of reduction in TNFα, IL-6, and NO protein *via* inhibition of the pro-inflammatory NF-κB and activation of the anti-inflammatory Nrf2 pathways (Lepiarz et al., 2017).

Furthermore, Ginkgo-biloba-derived have nanoencapsulated and demonstrated anti-inflammatory effects in Parkinson´s disease and arthritis models (Han, 2005; Zhao et al., 2020). Similarly, lycopene-loaded nanoparticles could reduce the knee-joint thickness, and inhibit the total leukocyte infiltration, the mononuclear cells, and the neutrophils in the inflammation site in arthritis *in vivo* models (Meira et al., 2020).

All these findings indicate the highly promising potential of nanostructured terpenes systems for inflammatory diseases.

## Discussion

In recent years, there has been a growing interest in natural compounds with therapeutic properties. Notably, as discussed in this article, numerous studies have demonstrated that terpenes and terpenoids possess a strong potential as alternative treatments for inflammatory diseases. Although not all the mechanisms of anti-inflammatory activity of terpenes have been described, it is known that these involve several molecular targets that include pro-inflammatory cytokines, transcription factors, autophagy machinery, ROS, membrane receptors, and other inflammatory mediators. Therefore, unlike some current drugs, terpenes can simultaneously act through different cell signaling pathways and exert a pleiotropic effect on inflammatory disorders; thus, terpenes could be more effective than existing medications.

On the other hand, despite the convincing evidence supporting the anti-inflammatory effects of terpenes, several concentration-dependent side effects should also be considered. Therefore, extensive investigations will be needed to evaluate their clinical efficacy and safety profile, which will allow establishing safe administration doses. In this regard, *in vitro* experiments cannot always be consistently replicated during *in vivo* studies. Likewise, information extracted from animal models cannot always be extrapolated to humans due to substantial inter-species variations; thus, controlled clinical trials will be crucial to establish the therapeutic effectiveness of these compounds. Finally, the medicinal effects of terpenes might be critically hindered by their low solubility and high instability; thus, their encapsulation in nanocarriers represents an attractive approach to complementing or replacing current medications.
